# Shape-shifting proteins and the subtlety in their form and function

**DOI:** 10.1016/j.isci.2026.117386

**Published:** 2026-09-08

**Authors:** Prakash Kulkarni, Supriyo Bhattacharya, Atish Mohanty, Sravani Ramisetty, Evan Pisick, John Orban, Vladimir Uversky, Keith Weninger, Sui Huang, Tsui-Fen Chou, Paul W. Sternberg, Ravi Salgia

**Affiliations:** 1 Department of Medical Oncology, City of Hope Medical Center, Duarte, CA, USA; 2 Department of Systems Biology, City of Hope Medical Center, Duarte, CA, USA; 3 Division of Biology and Biological Engineering, California Institute of Technology, Pasadena, CA, USA; 4 Department of Computational and Quantitative Medicine, Beckman Research Institute, City of Hope National Medical Center, Duarte, CA 91010-3000, USA; 5 City of Hope Chicago, 2520 Elisha Avenue, Zion, IL 60099, USA; 6 W. M. Keck Laboratory for Structural Biology, University of Maryland Institute for Bioscience and Biotechnology Research, Rockville, MD, USA; 7 Department of Chemistry and Biochemistry, University of Maryland, College Park, MD, USA; 8 Department of Molecular Medicine, Morsani College of Medicine, University of South Florida, Tampa, FL, USA; 9 Department of Physics, North Carolina State University, Raleigh, NC 27695, USA; 10 Institute for Systems Biology, Seattle, WA, USA; 11 Proteome Exploration Laboratory, Beckman Institute, California Institute of Technology, Pasadena, CA, USA

**Keywords:** shapeshifting proteins, dark proteome, allostery, intrinsically disordered Proteins, conformational dynamics, energy landscape

## Abstract

Shape-shifting proteins include intrinsically disordered proteins (IDPs) and fold-switching, or metamorphic proteins. They make up a significant fraction of the “dark proteome” in the protein universe. In this essay, we highlight some often misconceived or underappreciated features of shape-shifting proteins: First, the subtlety of the structure of IDPs; second, the relationship between intrinsic disorder and allostery, third, the possibility of discerning IDP conformational preferences based on energy landscape theory, and fourth, the contributions of disordered regions to fold switching in metamorphic proteins. Lastly, we highlight emerging evidence that fold-switching/metamorphic proteins may contribute to phenotypic plasticity and adaptive evolution by acting as evolutionary capacitors.

## Graphical abstract

**Figure:**
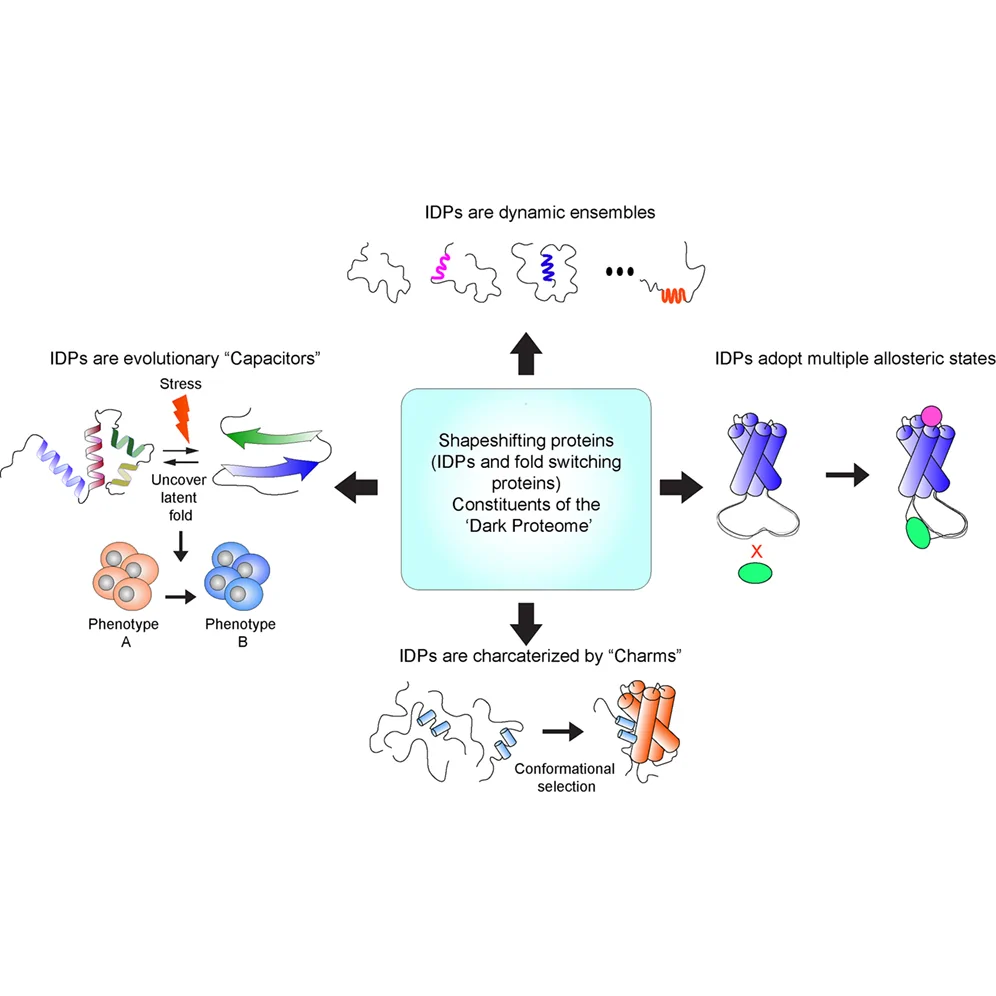

## Teaser

evolutionary biology; structural biology; biophysics

## Introduction

Within the vast complexity of the proteome in every organism, a remarkable class of proteins diverges significantly from the traditional sequence-structure-function paradigm (the protein sequence determines a stable, well-defined 3D structure governing biological function), namely the shape-shifting proteins. These include intrinsically disordered proteins (IDPs) and fold-switching proteins (also known as metamorphic proteins). Far from being rare exceptions, shape-shifting proteins constitute a significant fraction of the proteome known as the “dark proteome.” In addition, like the dark matter that constitutes a significant fraction of the universe’s total mass-energy density, the dark proteome points at a hidden layer of biological complexity, awaiting deeper exploration. The main reason that may account for the difficulty in fully comprehending the dark proteome is the lack of suitable techniques to probe proteins that seemingly lack structure as we know it.

Until the late 1990s, the protein universe was believed to be made up of only proteins that must have unique 3D structures if they were to be functional. The possibility that ordered proteins may contain regions that lack structure and yet be functional, although acknowledged already in the late 1950s, was largely ignored in light of the dominant one-to-one-to-one relationship between sequence, fold, and function of proteins.^1^^,^^2^^,^^3^ Likewise, the possibility that the same sequence can switch folds and acquire different functions was non-existent, simply because, at that time there was not a single example of an ordered protein that can switch between alternate folds and serve different functions.

The discovery of IDPs and the myriad functions they perform in every living organism was as intriguing as the proposal that matter can exist both as wave and particle by Louis de Broglie in the 1920s. How could a protein be functional if it lacked structure? After all, proteins were thought to exist in binary states: either structured, and hence, functional, or denatured, and therefore, non-functional.^2^ Even more surprising was the discovery in the late 1990s that the same (or similar) protein sequence can switch folds (fold-switching proteins) and assume different functions.^4^^,^^5^ These findings defied conventional wisdom that structure begets function and marked the beginning of a profound shift in our understanding of protein biology. As investigations continued to illuminate this hidden proteomic frontier, they revealed that disorder, far from being the mere absence of structure, is a fundamental and finely tuned feature of life’s molecular machinery. Nonetheless, it is not our intent to preach to the choir since several excellent reviews/perspectives have been published on this very topic.^3^ Instead, our main purpose here is to highlight a few fundamental features of shape-shifting proteins that uncover the subtlety in their form and function.

## IDP structure and clairvoyance

Our first point concerns the perception of “structure” (or the lack of it) in IDPs. Unfortunately, it is often misconceived that IDPs *lack* structure; yet, they are functional, and therefore, they present a *challenge* to Anfinsen’s dogma.^6^^,^^7^ However, this interpretation reflects a limited understanding of what “structure” truly entails in the context of IDPs. To dispel this reductionist view, it may be helpful to adopt a metaphorical lens, thinking about IDP structure not through the conventional framework of fixed atomic coordinates, but more like a surrealist would perceive it.

**Figure 1:**
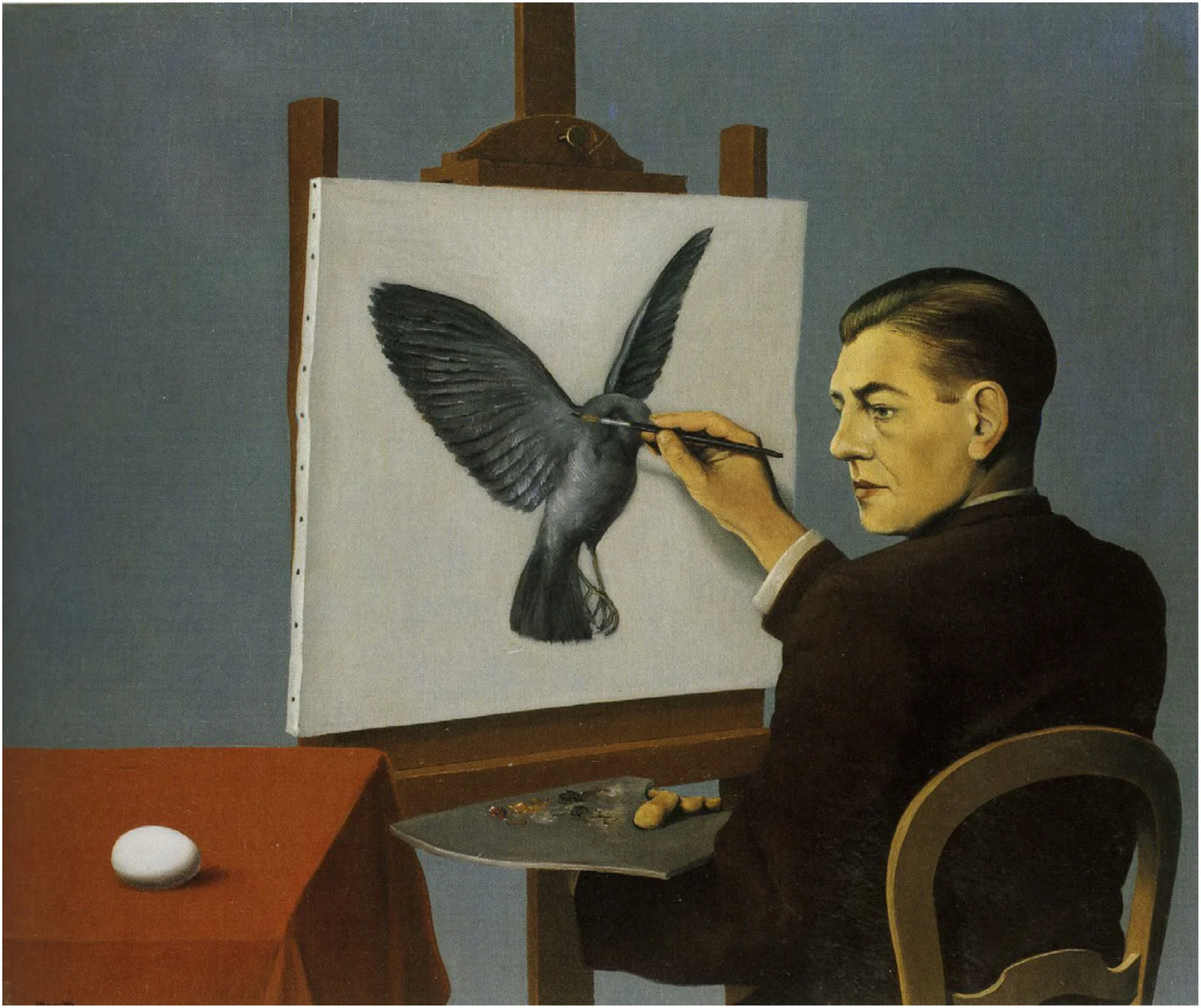
René Magritte. La Clairvoyance (Clairvoyance). Brussels, 1936 Source, The Museum of Modern Art (“MoMA”) https://www.moma.org/audio/playlist/180/2391.

Surrealist artists from the early 20th century were known for expressing ideas in unconventional, boundary-defying ways to reveal deeper truths. *La Clairvoyance* (1936), a self-portrait by the Belgian surrealist René Magritte (Figure 1) is a good case in point. In this self-portrait, Magritte is seen painting a fully developed bird although he is gazing at a bird’s egg as the model, a striking rendering that suggests an imaginative leap between what is observed and what is perceived. Thus, a surrealist frame of reference may help better appreciate IDPs, namely not by denying structure, but by acknowledging that structure might manifest in dynamic, probabilistic or latent ways that escape the traditional structural biology’s lens. Like Magritte’s surreal art, the relationship between an IDP ensemble and its function may require a conceptual leap, a realization that what is functionally relevant need not always be immediately visible or classically “structured.” The painting inspires one to recognize that what appears amorphous or disordered may in fact be governed by nuanced principles. Phenotypes and other biological phenomena linked to IDPs are often shaped by details in their conformational dynamics that may be hidden from plain sight and yet are critical to function. It is, therefore, not surprising that IDPs serve as critical components of the cell’s physical wetware that enables the cell to “make” decisions emanating from the software (genome-encoded sequences). Below we present two specific examples of cellular decisions to appreciate how IDPs influence them.^8^

The above framework of abstraction becomes especially powerful when examining specific examples of disordered proteins such as the cancer/testis antigen prostate-associated gene 4 (PAGE4). PAGE4 that is expressed in androgen-dependent prostate cancer epithelial cells, is a highly (>90%) IDP, and thus, is present as a statistical ensemble of molecules in distinct conformations. PAGE4 is a stress-response protein and a transcriptional regulator that potentiates another regulator, namely the oncoprotein c-Jun, which dimerizes with the oncoproteins c-Fos to form the AP-1 transcription factor complex. However, not only the activity of PAGE4 but also the conformational dynamics are regulated by two kinases, namely HIPK1, which compacts the PAGE4 ensemble and turns PAGE4 on, and CLK2, which relaxes the structure to resemble an ensemble of random coils thus expanding dynamics and diversity of the ensemble of PAGE4 molecules, and turns PAGE4 off. Therefore, in addition to the activity of a protein, here, the regulation by the kinases also affects a statistical property of the stochastic ensemble of the molecule.

**Figure 2:**
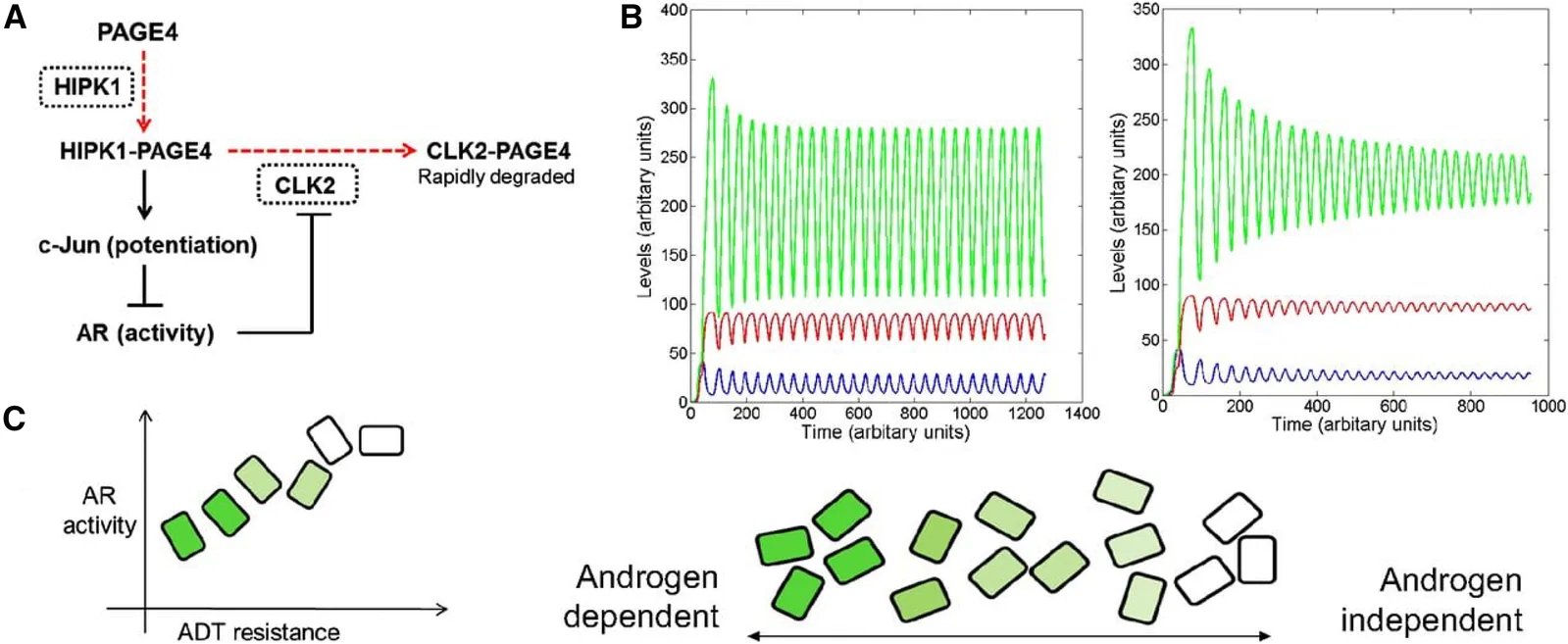
Modeling the PAGE4/AP-1/AR/CLK2 regulatory circuit (A) Regulatory circuit for PAGE4/AP-1/AR/CLK2 interactions. Dashed red lines denote enzymatic reactions, and solid black lines denote nonenzymatic reactions. CLK2 and HIPK1, the two enzymes involved, are shown in dotted rectangles. (B) Dynamics of the circuit showing sustained and damped oscillations for HIPK1-PAGE4 (PAGE4_M_, shown in blue), CLK2-PAGE4 (PAGE4_H_, shown in red), and CLK2 (shown in green). Parameters are given in Figure S9.^9^ (C) Distribution of androgen dependence for an isogenic population over a spectrum, as indicated by the shade of green. Dark green boxes denote highly androgen-dependent (i.e., ADT-sensitive) cells, and white boxes denote androgen-independent cells. Reproduced from reference.^9^

Mathematical modeling of the PAGE4 regulatory circuit, which considers this alteration of ensemble statistics by the two kinases, revealed synchronous oscillations in its components; specifically, in PAGE4, HIPK1, and CLK2. The oscillatory dynamics has been proposed to help maintain the epithelial cell’s androgen dependence. However, dampening oscillations renders the cell independent of androgen.^9^ Thus, the decision of a prostate cancer epithelial cell to either remain androgen dependent or become independent and metastasize to distant locations is actuated by the conformational dynamics of PAGE4. Consistent with this observation, in a population of isogenic androgen-dependent prostate cancer cells, there exist cells that can stochastically sample the androgen-independent phenotype space (randomly and transiently become androgen-independent), suggesting a bet-hedging strategy to enhance fitness of the isogenic population in dynamic environments through phenotypic heterogeneity (Figure 2).^10^^,^^11^

Further, in castrate-resistant prostate cancer wherein epithelial cells are androgen independent, PAGE4 expression is shut off, presumably by epigenetic modifications. However, intermittent androgen-deprivation therapy restores expression and oscillations in the PAGE4 circuit.^12^ It is possible that the absence/presence of androgen (intermittent therapy) causes changes in the DNA regulatory region(s) of PAGE4 to impact its expression in response to the environment. The fact that the expression of several cancer/testis antigens including members of the PAGE family that is undetectable in prostate cancer cells but is inducible by treatment with epigenetic modifiers such as 5-Aza-2′-deoxycytidine (5AZA) and/or a histone deacetylase inhibitor LBH589, lends further credence to the hypothesis of epigenetic control.^13^

Together, these findings illustrate how the abstract principles of conformational flexibility, gene regulatory plasticity, and environmental sensing coalesce in the biology of PAGE4. They reinforce the broader notion discussed above: that IDPs, far from being structurally irrelevant, operate as dynamic integrators of cellular context.

**Figure 3:**
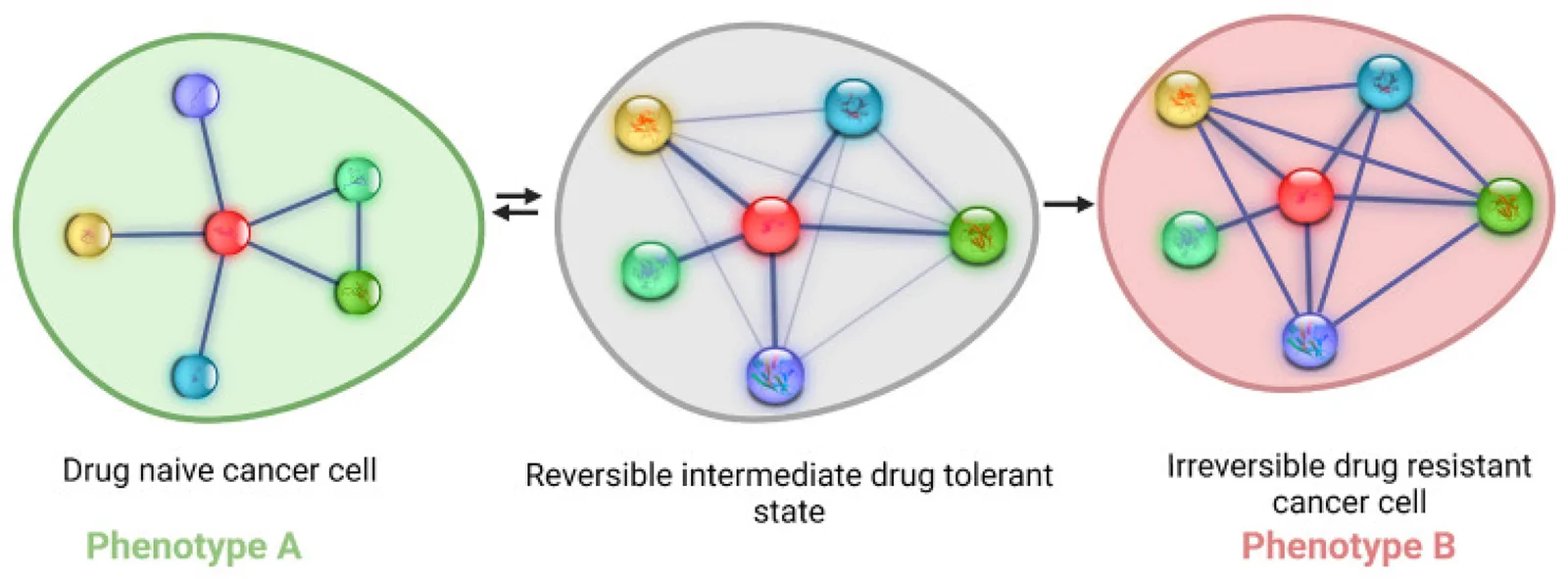
Phenotypic switching from drug-sensitive to drug-resistant phenotype via protein interaction network (PIN) rewiring Upon exposure to a drug, sensitive cells phenotype A, attempt to evade the harmful drug effects by rewiring their PIN. This non-genetic mechanism results in a reversible drug-tolerant state. However, prolonged drug treatment leads to network frustration, which leads mutations in certain “hot spot” loci to alleviate this frustration. This results in the emergence of an irreversible drug-resistant state, referred to as phenotype B. Reproduced from ref.^14^

A second example of cellular decision-making concerns the emergence of drug resistance, especially in the context of cancer (Figure 3). Irreversible drug resistance can pre-exist and can be due to genetic mutations (innate drug resistance). Nonetheless, it can also arise in response to drug treatment (acquired resistance) and can be driven by both genetic and non-genetic mechanisms. In the latter case, the system rewires its protein interaction network (PIN) that involves IDPs occupying key hub positions in the network, to develop tolerance to evade the harmful drug effects.^15^^,^^16^ This rewiring is reversible and takes place through a combined effect of epigenetic changes and post-translational modifications (PTMs).^17^^,^^18^^,^^19^ The targets of PTMs are often IDPs or disordered regions of folded proteins, due to their sensitivity toward “shifting shapes” (alter conformation) in response to PTMs such as phosphorylation.^20^ Upon withdrawal of the drug treatment, the system reverts to being drug sensitive from the drug tolerant state. This reversal highlights the non-genetic mechanism underlying drug tolerance. However, chronic drug treatment can lead to “frustration” of the PIN that is relieved by mutations that “fix” the drug-resistant phenotype.^14^ It remains debatable whether these mutations happen in specific locations consistent with promoting the induced resistant phenotype, which may be more prone to DNA damage; or, subsequent mutations occur due to transcriptional activity (exposing open DNA) (mutational hotspots) and represent suitable targets for powerful selection. Regardless, this results in irreversible, genetically specified resistance to the drug.^21^^,^^22^^,^^23^ Thus, the cell’s “decision” to evade the toxic drug effects employing this two-step strategy highlights the genetic/non-genetic duality in Nature and reveals an evolutionary bet hedging strategy to maximize survival in the event of an unanticipated calamity without depending solely on the rare random “adaptive mutations” of the neo-Darwinian scheme.^11^^,^^18^^,^^24^ In both the above examples, one cannot fail to be impressed by how IDPs play a critical role in the way the phenotype rapidly responds to changes in the environment or, to miss noting that the decisions between phenotypic fates that a cell makes can be independent of genome alterations, that is, these decisions are not dictated by the DNA sequence but by the information encoded in IDP’s statistical ensemble.

In fact, one begins to appreciate that contrary to common wisdom, it is possible, perhaps even plausible, that under certain circumstances, shape-shifting proteins can transmit information from the phenotype to modify the genotype for transgenerational inheritance.^25^^,^^26^^,^^27^^,^^28^^,^^29^^,^^30^^,^^31^^,^^32^^,^^33^ A causal link between epigenetic changes in response to changes in environment, dysregulation of an IDP, namely bone morphogenetic protein 4, and the evolution of the beak of Darwin’s finches by natural selection on the Galapagos Island, maybe another good example of how information is reverse transferred to the genotype.^34^ Although the subject of a raging debate between geneticists favoring natural selection of random mutations (Darwinian evolution) and the naturalists defending inheritance of acquired characteristics (Lamarckian induction), the possibility of reverse information flow from phenotype to genotype, and the subsequent inheritance of such acquired characteristics, is compelling in view of the oft unfathomably rapid evolutionary innovations. It calls for reconciling evolution as being both Darwinian selection and in some way, Lamarckian induction. This possibility awaits mechanistic elaboration, which will involve epigenetic and cytoplasmic state and cytoplasmic inheritance, now further expanded to include shape-shifting proteins, and does not undermine the importance of random genetic mutations in evolution.

## Intrinsic disorder and allostery

Our second point concerns intrinsic disorder and allostery. Historically, allosteric regulation was the first officially recognized and broadly accepted concept directly related to the very idea that proteins undergo shapeshifting. Allostery refers to the process where the binding of a regulator molecule (ligand) to one site on a protein affects the function of a different, distant site on that same protein, e.g., enzymatic activity. This is an important property of proteins in the regulation of a diversity of biological functions, such as catalysis, molecular recognition, signal transduction and transport. Although the allosteric effect model was proposed in 1961 by Monod and Jacob to describe an inhibitory action of a ligand that is not sterically analogous to the substrate, the idea that binding of one molecule to a protein can affect its binding affinity to another molecule was known since 1904, when Bohr, Hasselbalch and Krogh described how carbon dioxide affects oxygen binding to hemoglobin in blood, a phenomenon known as the Bohr effect.^35^^,^^36^

**Figure 4:**
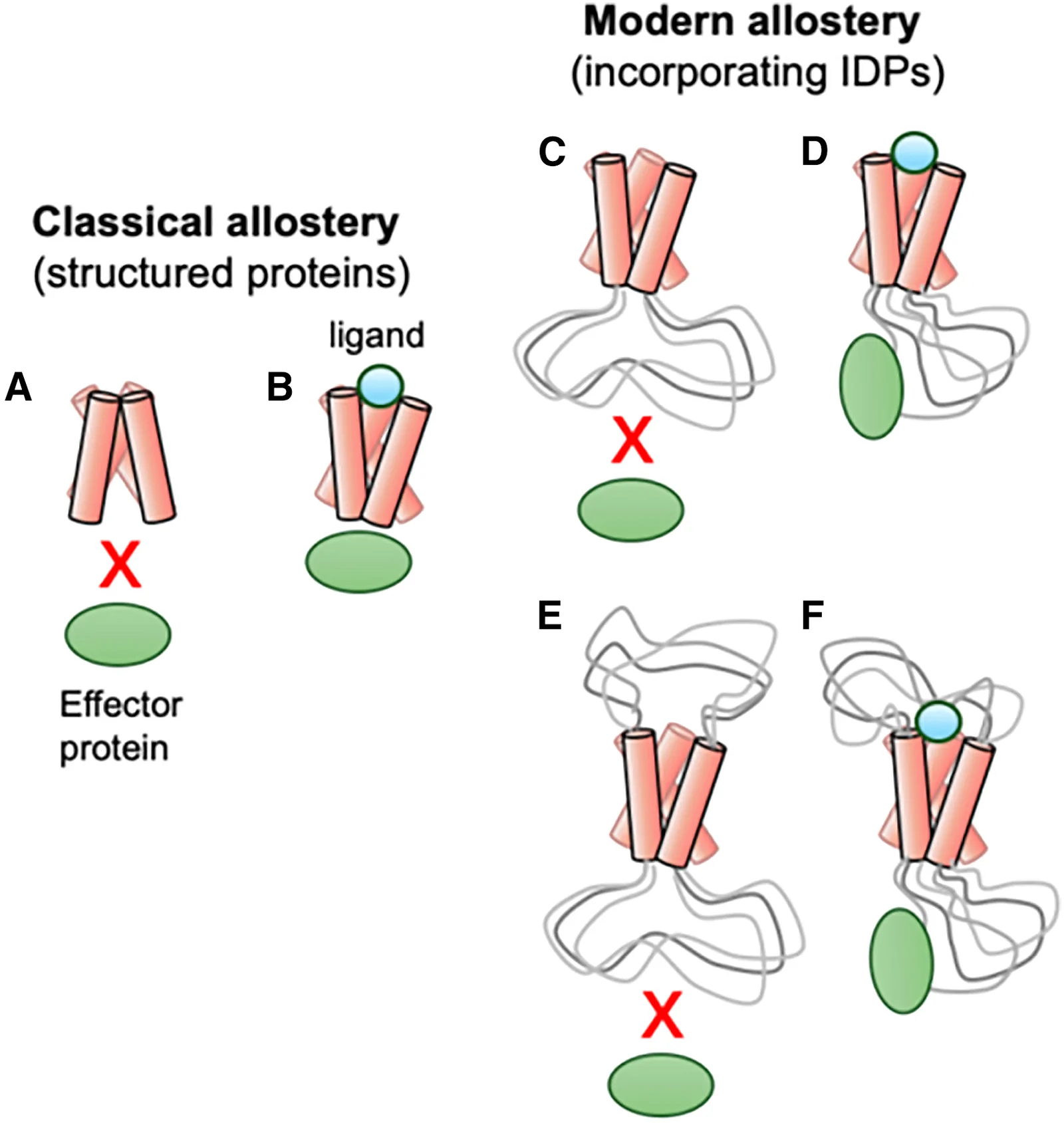
Schematics describing classical vs. modern views of allostery incorporating IDPs Schematics describing classical (A and B: structured proteins only) vs. modern views of allostery (C–F) incorporating IDPs. In the classical model, allostery is propagated via conformational changes in structural domains where binding of ligand at one site would trigger mechanical changes at a distant site (often through sequential domain movements) allowing effector proteins to bind (A and B); In the modern view, allostery can be propagated through thermodynamic coupling between distant domains, with little conformational changes in the structured domains that serve as conduits (C–F). This form of allostery is facilitated by the dynamic ensemble changes in disordered regions at one (C and D) or both ends of the allosteric conduit (E and F).

Over the years, the concept of the allosteric effect evolved from two classic models, namely the concerted Monod-Wyman-Changeux model and the sequential Koshland-Nemethy-Filmer model as explained below.^37^^,^^38^^,^^39^ Both assume conformational change in a two-state model of dynamic allostery in the ensemble model, where allosteric communication between distinct binding sites is established via ligand-induced changes in protein dynamics (Figures 4A and 4B).^40^ Originally considered as applicable to multi-domain and multi-subunit proteins, allostery was eventually extended to all proteins.^38^^,^^39^^,^^41^ The importance of shapeshifting in allostery was recognized already in 1965, when it was pointed out that “*By their very nature*, *allosteric effects cannot be interpreted in terms of the classical theories of enzyme action*. *It must be assumed that these interactions are mediated by some kind of molecular transition (allosteric transition) which is induced or stabilized in the protein when it binds an ‘allosteric ligand’*.”^38^

More recently, an important correlation between allostery and intrinsic disorder was pointed out, with intrinsic disorder acting as a mechanism for the optimization of allosteric coupling in proteins. Here, intrinsic disorder in the domains or segments present in either one or both of the coupled binding sites, appears to maximizes site-to-site allosteric coupling (Figures 4C–4F).^42^ Furthermore, it was recognized that because of all these changes, the allostery concept became fuzzy over time.^43^ In fact, “*proteins defined to be allosteric include not only those that obey the two-state concerted model*, *but also those that obey different reaction mechanisms*, *such as ligand-induced fit*, *possibly coupled to sequential structure changes*, *and ligand-linked dissociation-association*. *Since each reaction mechanism requires its own mathematical description and is defined by it*, *there are many possible ‘allosteries’*.”.^43^

In the previous two decades, multiple computational and biophysical studies have shed light on allosteric mechanisms in disordered proteins.^42^^,^^44^^,^^45^^,^^46^ It is now widely recognized that disordered domains act as important conduits for allosteric signal that are critical for cellular function in processes such as cell cycle and immune signaling.^47^ Due to their dynamic nature, mechanistic description of allostery in IDRs remains elusive. However, several mechanisms have been proposed such as the ensemble allosteric model (EAM) that provides a thermodynamic basis of energetic coupling between distant protein sites through the conformational ensemble of IDRs.^42^ Based on EAM, the authors explained allosteric regulation in human glucocorticoid receptor, where a small subset of protein conformations elicit transcriptional activation or repression through energetic coupling of distant domains in response to ligand or co-factor binding. Without any stimulus, the receptor is maintained in the inactive state through energetic “frustration” (see subsequent section for discussion on frustration) mediated by the IDR.^46^ Besides ligand binding, PTM sites (that are deeply concentrated within disordered domains) constitute an important source of allosteric modulation by IDRs.^20^^,^^48^^,^^49^ An example of PTM driven allostery is the cell cycle regulator p27^Kip1^, where one tyrosine phosphorylation cooperatively induces additional phosphorylation, some of them at distant sites to regulate the interaction with the Cdk2/cyclin A complex.^50^ PTMs in IDRs (and subsequent allostery-driven signal amplification) also play a critical role in the activation of T cell receptors, and by extension crucial for the efficacy of CAR-T therapy.^51^ In summary, allostery in IDRs/IDPs is a complex phenomenon, harnessing protein dynamics and thermodynamic principles of cooperativity and frustration.^52^ The diversity of structural ensembles of IDRs translates into the potential for multiple allosteric states adopted by a single protein, thereby supporting the role of IDPs as functional cellular hubs.

## Discerning IDPs based on their conformational preferences

Our third point is that, just as ordered proteins are made up of differing secondary structure, intrinsic disorder in proteins may also be made up of distinguishable types, comprising members with distinct and usually dynamic Ramachandran angles. If so, can IDPs be described as having unique characteristics that group into categories, analogous to how the fundamental particles of nature can be categorized based on shared characteristics like being fermions or bosons (distinguished by integer or non-integer spin quantum number), being massive or massless, etc.? Indeed, Vucetic et al. developed an algorithm that partitions protein disorder into different “flavors,” namely the V, C, and S flavors, that can distinguish IDPs based on amino acid composition, sequence locations, and biological function.^53^ Overall, their results are complementary to structural genomics, as they appear to support the flavor-function approach in automatically assigning possible functions to IDP sequences. However, there are no data available that suggests that flavors can predict the conformational preferences/dynamics of IDPs before/after interacting with cognate partners. Besides the aforementioned scheme, several proposed classification schemes for IDPs include those based on function (e.g., chaperones, macromolecular assemblers, or scavengers), functional motifs (e.g., proteolytic cleavage or phospho-sites), evolutionary conservation and biophysical properties such as solubility.^54^ Each of these schemes focuses on one aspect of IDP modality and it’s still not entirely clear how one classification scheme relates to another. Thus, no consensus classification scheme exists that considers IDP behavior holistically. Physics based principles (e.g., those embedded in the energy landscape theory) bear the potential to unite apparently heterogeneous IDP behavior by analyzing the fundamental forces that govern their sequence → ensemble → function relationships.

**Figure 5:**
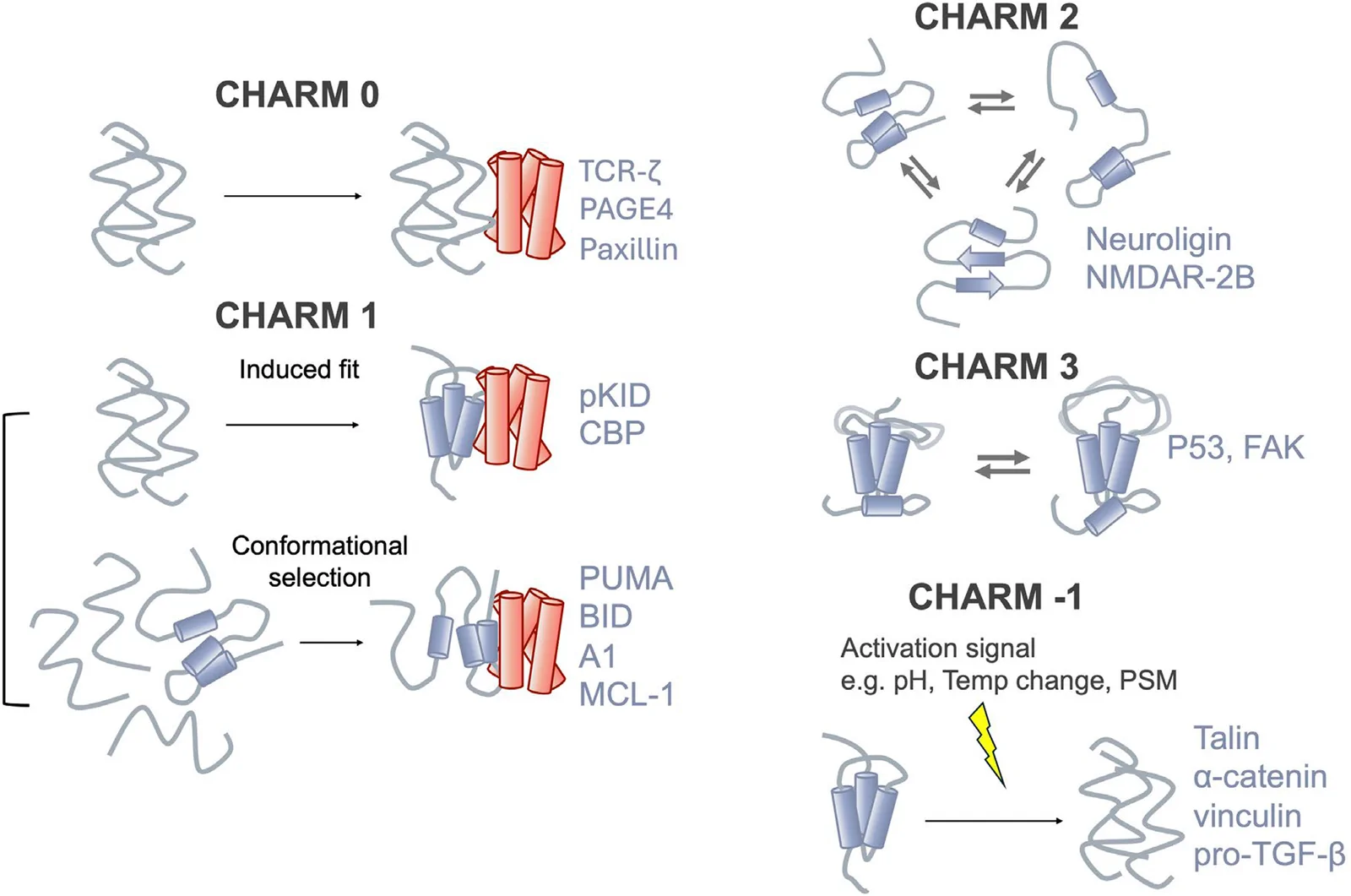
Diagrammatic representation of the various IDP charms IDRs, helices and beta sheets are represented by coils, cylinders and block arrows respectively. IDPs are shown as gray, and binding partners in orange. For each charm category, example proteins are listed to the right.

We suggest that it may be possible to determine the probability of an IDP’s conformational preferences independent of its V, C, and S flavor by employing the energy landscape theory. Unlike well-folded proteins, the energy landscape of IDPs can be different for different IDPs since they do not have a single, stable tertiary structure. IDPs exist as ensembles of rapidly interconverting conformations. Hence, the energy landscape, which describes the potential energy of these different conformations, can vary significantly between different IDPs due to differences in their amino acid sequences and the interactions they can form. Furthermore, it has also been observed that IDPs can behave like entirely different polymers in different regions of the conformational space.^55^ Therefore, we reason that IDPs may be characterized by another property we term “charms” to describe their conformational preferences in the free states (the apo form) (Figure 5). The inspiration for this terminology comes from particle physics, where the charm quantum number represents the quark compositions classifying different subatomic particles. Similar to particles with varying “charms” behaving differently, IDPs can show varying conformational preferences and dynamic behavior depending on which charm they belong to (Table 1). The following examples of charm-based classification of IDPs are based on data reported in the literature that were obtained experimentally or by employing physics-based computational methods or both.

**Table 1:** The various IDP charm categories, their physical landscape characteristics, and representative protein examples

Charms	Characteristics	Examples
Charm 0	These IDPs exhibit no or little discernible conformational preferences and can interact with cognate targets while remaining disordered.	C-terminal tail of the histone H1, prothymosin-α, cytoplasmic domain of the ζ chain from the T cell receptor, PXN and PAGE4.
Charm 1	These IDPs can randomly sample the conformational space due to thermal motion in the absence of an interacting partner (enabling conformational selection) or can do so only in the presence of the interacting target (consistent with the induced fit model).	Disordered BH3-only proteins such as PUMA and BID and the folded BCL-2-like proteins A1 and MCL-1 (conformational selection). Phosphorylated kinase-inducible domain (pKID) of the cAMP-response element binding (CREB) protein, and the KIX domain of a general transcriptional coactivator CREB-binding protein (CBP) (induced-fit mechanism).
Charm 2	IDPs that exhibit strong conformational preferences and hence, sample a restricted search space. They are the so-called hop-switch type IDPs. They undergo conformational excursions to stochastically switch among a limited set of distinct conformational states.	Neuroligin and the NMDAR-2B glutamate receptor.
Charm 3	These IDPs are typically seen in folded proteins where the conformational dynamics of the entire protein molecule is sensitive to conformational changes in the disordered regions.	p53 and FAK.
Charm −1 (or anti-charm)	IDPs embody those proteins that transition from order to disorder (rather than disorder to order) to become functional, Negative sign denotes the reverse transition.	Talin, α-catenin, vinculin, and prodomain transforming growth factor β (pro-TGF-β).

To start, we associate charm 0 IDPs with those IDPs that exhibit no or little discernible conformational preferences and can interact with cognate targets while remaining disordered. The C-terminal tail of the histone H1 and its nuclear chaperone prothymosin-α, cytoplasmic domain of the ζ chain from the T cell receptor, paxillin (PXN) and PAGE4 are good examples.^9^^,^^56^^,^^57^^,^^58^ Although a quantitative estimate of the global prevalence of Charm 0 type IDPs is lacking at the moment, it is possible that it may constitute a significant fraction based on the growing list of IDPs that retain conformational heterogeneity or structural plasticity in the bound state, forming the so-called “fuzzy complex,” rather than undergoing folding upon transition to binding.^59^^,^^60^^,^^61^ Furthermore, based on the concept of protein frustration, which occurs when a physical system such as a protein is unable to simultaneously achieve minimum energy individually for each and every part of the system, it was discovered that IDP complexes exhibit a high degree of local frustration, especially at the binding interface, leading to multiple suboptimal interactions. Interestingly, the multiple bound substates appear to have distinct frustration patterns when they interact with different partners. This may help explain how specificity of IDPs is achieved in the absence of a unique common bound conformation and how competition between different interactions can dictate the binding to multiple partners.^62^

Recent observations on PXN highlight how IDRs in charm 0 type IDPs contribute to the conformational dynamics of the entire molecule. PXN is a major component of the focal adhesion complex, a multiprotein structure that links the intracellular cytoskeleton to the cell exterior. In this complex, PXN interacts with the C-terminal targeting domain of focal adhesion kinase (referred to as FAT) via the leucine-rich LD motifs in N-terminal domain. Other than the 5 LD motifs (LD1-5) that are each made up of ∼12 amino acids and are helical, the regions between the LD motifs is highly intrinsically disordered. Upon interacting with FAT, although the PXN N-terminal region remains intrinsically disordered, it undergoes large-scale conformational restriction with four major interconverting states that determine the orientation of the PXN molecule when interacting with FAT. Since PXN acts as a hub protein in the interaction network that establishes the focal adhesion complex, these observations provide new insight on PIN rewiring in cellular mechanotransduction in response to environmental perturbations.^58^ Such IDP-mediated network rewiring can have significant biological consequences such as phenotypic switching.

Charm 1 IDPs include those that can randomly sample the conformational space due to thermal motion in the absence of an interacting partner (enabling conformational selection) or that can do so only in the presence of the interacting target (consistent with the induced fit model). The disordered BH3-only proteins PUMA and BID and the folded BCL-2-like proteins A1 and MCL-1 represent IDPs that follow the conformational selection paradigm for partner interaction, while phosphorylated kinase-inducible domain of the cAMP-response element binding (CREB) protein with the KIX domain of a general transcriptional coactivator CREB-binding protein are good examples of the induced-fit mechanism.^63^^,^^64^

Charm 2 IDPs are those that exhibit strong conformational preferences and hence, sample a restricted search space. They are the so-called hop-switch type IDPs. They undergo conformational excursions to stochastically switch among a limited set of distinct conformational states. Neuroligin and the NMDAR-2B glutamate receptor are IDPs with charm 2 characteristics.^65^

By contrast, charm 3 IDPs (IDRs) are typically seen in folded proteins where the conformational dynamics of the entire protein molecule is sensitive to conformational changes in the disordered regions. Examples include p53 and FAK.^66^^,^^67^^,^^68^^,^^69^

Finally, anti-charm (or charm −1) IDPs embody those proteins that transition from order to disorder (rather than disorder to order) to become functional. Examples include talin, α-catenin, vinculin, and prodomain transforming growth factor β (pro-TGF-β).^70^ The negative sign denotes the reverse transition. The chaperonin machines and protease machines, where the protein substrate gets unfolded for the purpose of subsequent refolding or shredding, may represent additional examples of anti-charm IDPs.^71^^,^^72^

**Figure 6:**
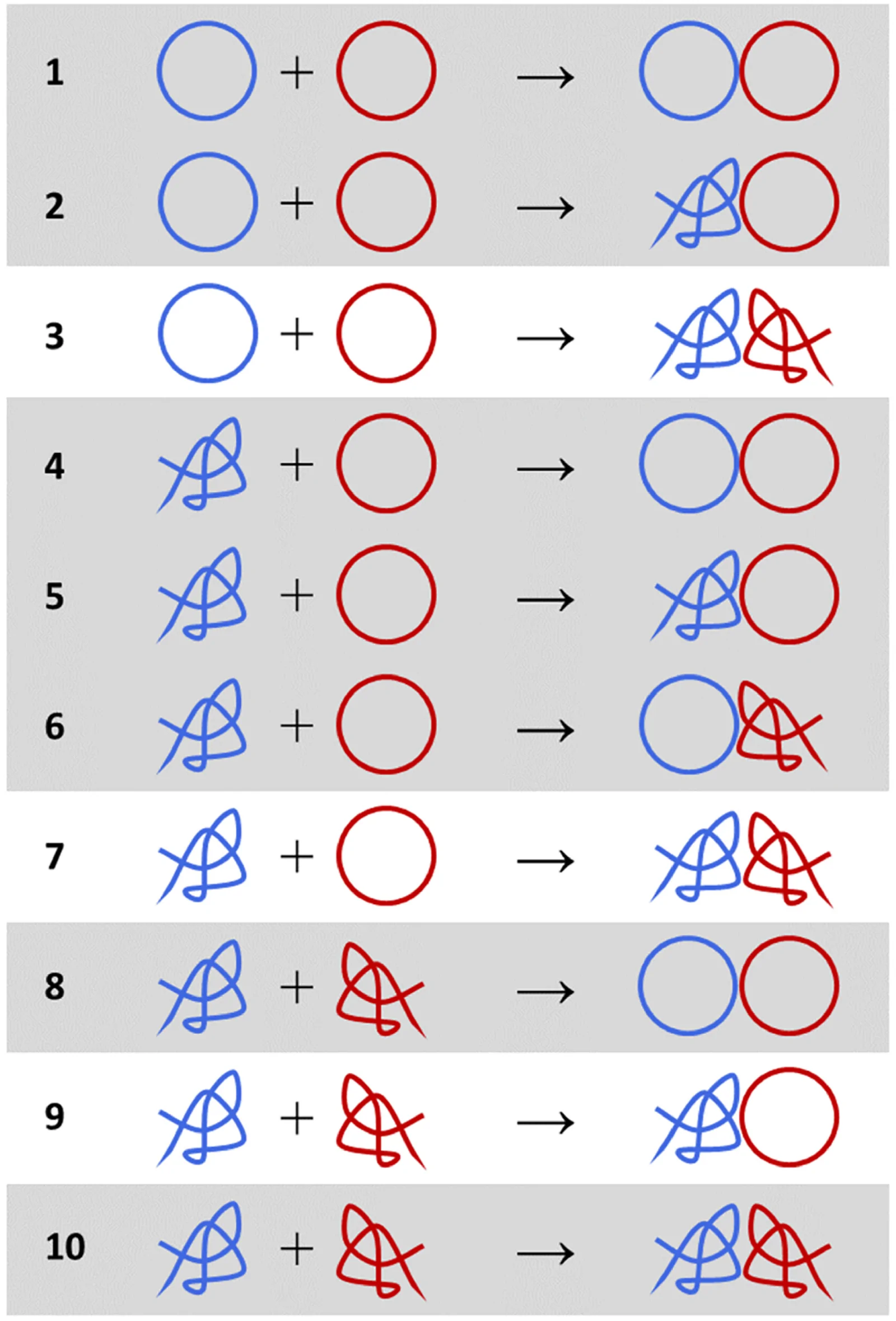
Different variants of order-disorder transition upon protein binding Those scenarios that have been observed experimentally are indicated by gray shading. The representation is limited in that it refers only to the initial and final states of the system and does not address the mechanism of binding. For example, scheme 4 may involve conformational selection or induced fit or combination thereof. Reproduced from reference.^73^

It is generally held that the free energy landscape of IDPs is almost flat, such that all conformations are of roughly equivalent energy and the transition barriers are small. However, it is now increasingly evident that this assumption may not be true for all IDPs. In some cases, the height of the transition barriers may be non-uniform and as a result, intramolecular diffusion in conformation space can become transiently restricted in localized free energy minima resulting in the different behaviors observed (Figure 6). With advances in prediction and characterization of IDP structural ensembles, it is likely that IDPs can be systematically characterized with respect to their landscapes and hence, categorized by different charms.^74^^,^^75^ Some IDPs may behave differently (with different charm) in response to changes in their environment. Furthermore, it is entirely possible that additional IDP charms may come to light for categorizing order/disorder transitions as new discoveries are made if one considers protein-protein interactions (Figure 5 where ordered proteins are indicated as red/blue circles while disordered proteins are denoted by red/blue squiggles).^73^ Additionally, with the advances in employing machine learning and artificial intelligence in computing IDP conformational dynamics, it may not be a leap to suggest that soon it would be possible to predict their behavior in the presence or absence of an interacting partner, *a priori*. Such information can be extremely helpful in targeting IDPs that play important roles in disease states. Furthermore, given the dramatic variation in the length of the resident states of IDPs, they are more than likely to contribute significantly to (conformational) noise which translates into stochasticity of interactions, which in turn can have significant biological implications.^76^

## Fold-switching/metamorphic proteins with disordered regions

**Figure 7:**
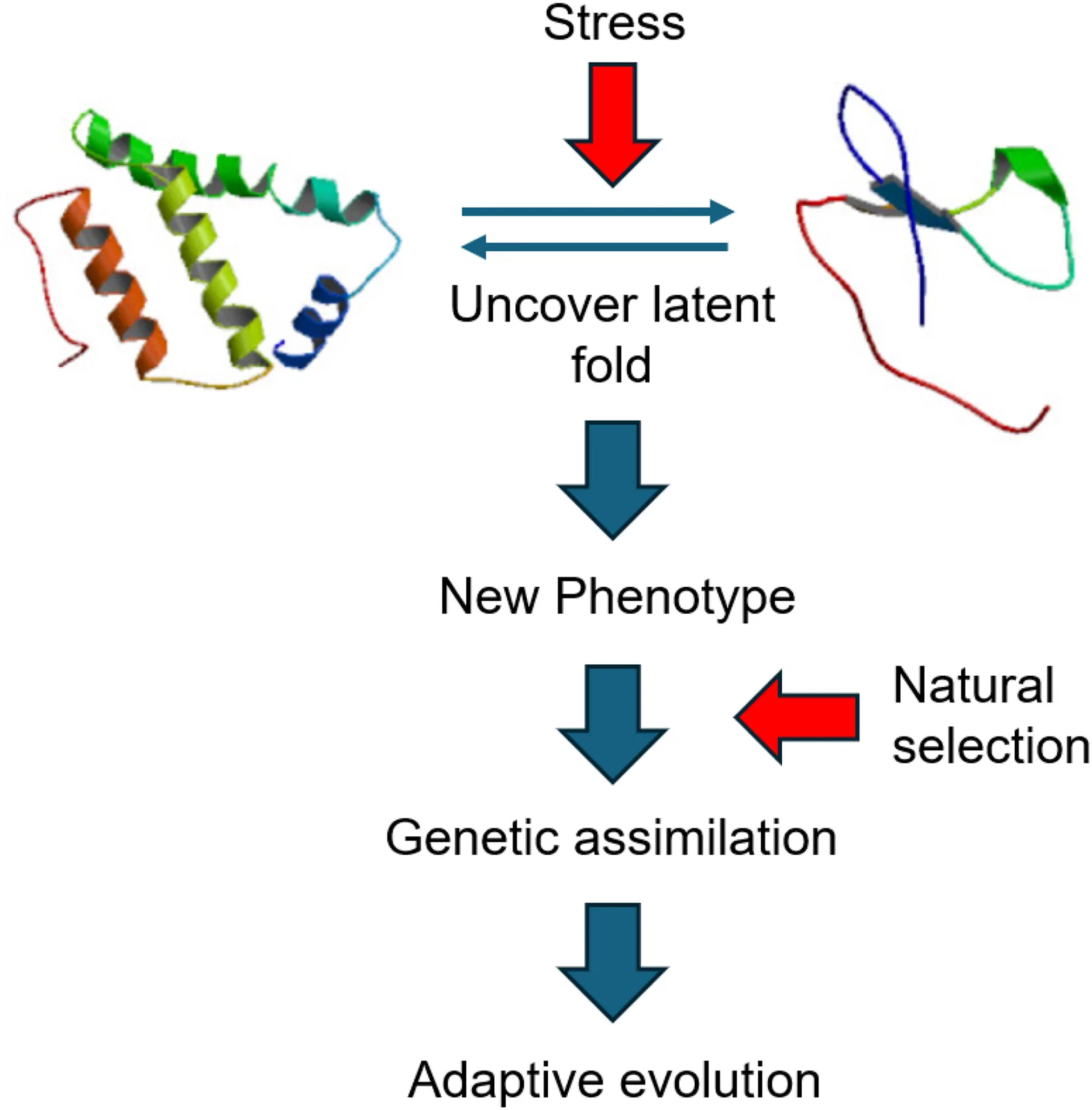
Fold-switching proteins as evolutionary capacitors

Our fourth comment seeks to draw attention to the role of fold switching proteins in phenotypic plasticity. Living systems are robust to mutations and can accumulate genetic variations that remain cryptic, i.e., they do not have a phenotypic effect. But when the system is perturbed (e.g., by stress), robustness breaks down, and cryptic variants are exposed, causing phenotypic effects which are subject to natural selection.^77^^,^^78^ A striking example of the case in point is the 4-winged fruit fly. Waddington generated the latter by exposing flies at the appropriate developmental stage to ether vapor that apparently did not cause genetic mutations.^79^ Instead, as Waddington explained, environmental stress helped uncover cryptic variants in the population. The exposed variants were then subject to natural selection and, eventually, genetic assimilation, i.e., the fixation by a mutation of the realization of the cryptic variant without environmental stress (ether vapor). Thus, it is tempting to speculate that like the molecular chaperon HSP90, fold-switching/metamorphic proteins may act as evolutionary capacitors that can switch genetic variations between hidden and revealed states, and in doing so, facilitate adaptive (Darwinian) evolution (Figure 7). Given the presence of a large number of genetic variants in a population, many apparently cryptic, such a mechanism appears plausible.

The cyanobacterial protein KaiB that plays a crucial role in regulating the circadian clock and compensates for changes in ambient temperatures, may serve as another illuminating case. Circadian clocks help to provide a reliable internal representation of time over a wide range of physiological temperatures through a compensation mechanism. The low-temperature thioredoxin-like fold in KaiB is stabilized by interacting with KaiC (the environmental trigger). In the absence of KaiC, homo-tetramerization of KaiB helps stabilize the unique and more well-packed high-temperature fold. The temperature-dependent equilibrium of the 2-folds serves to compensate for changes in ambient temperatures. This raises the intriguing possibility that fold-switching proteins, such as KaiB that help in the adaptation of the organism to changes in ambient temperature, at least in the case of ectotherms, may contribute significantly to adaptive evolution, especially in rapid evolutionary radiations.^80^^,^^81^

Other examples of fold-switching proteins driving phenotypic plasticity include (i) chloride intracellular channel 1 (CLIC1), which is known to promote cell cycle progression and cancer stem cell renewal, (ii) the microtubule-associated protein tau, and (iii) the myocyte-specific enhancer factor 2 B or MEF2B.^82^^,^^83^^,^^84^ CLIC1 is also known to promote migration and metastasis, suggesting a role in switching phenotypes to a more mesenchymal phenotype.^85^ However, how the alternate folds of CLIC1 actuate phenotypic switching has not been elucidated. The etiology of several neurodegenerative diseases, including Alzheimer’s, is thought to be due to the conversion of proteins into pathogenic conformations. For example, tau converts into β-sheet-rich amyloid conformations, which underlie pathology in over 25 related tauopathies. Indeed, biophysical studies of tau amyloid fibrils from diseased tissues revealed that tau adopts diverse structural polymorphs, each linked to a different disease.^83^ MEF2B is a major target of somatic mutations in non-Hodgkin lymphoma. A particular mutation, D83V that is frequently encountered in the disease, induces a dramatic α-helix to β-strand switch in the MEF2 domain. Indeed, a number of well-known targets in cancer harboring β-strand favoring mutations in chameleon α-helices have been observed, highlighting a commonality of such conformational switching in cancer.^84^ Together, these examples provide support for the involvement of fold-switching proteins in phenotypic plasticity.

## Conclusions

In this perspective, we have tried to bring to fore certain aspects of shapeshifting proteins that have not been fully appreciated, much less well studied. We trust that by highlighting these aspects including the extremely subtle nature of the IDP ensemble form/function, the possibility that they can be discerned based on their conformational preferences, the intriguing link between ensemble dynamics and allostery, and their role in phenotypic plasticity, will inspire additional investigations going forward.

Even though still a contentious issue, phenotypic plasticity, especially adaptive phenotypic plasticity, appears to play an important role in adaptive evolution.^86^^,^^87^ It is also believed to speed up evolution. For example, a computational study showed that when a genetic mutation causes a population to become less fit, (non-genetic) switching to an alternative phenotype with higher fitness (growth rate) may give the population enough time to develop compensatory mutations that increase the fitness again. Although this process is based on an *in-silico model* the study does offer deeper insight into real world scenarios. The best-known example is the emergence of antibiotic-resistant bacteria. It is possible that switching to a slower growing but less drug-sensitive phenotype, known as persister phenotype that exhibits increased drug tolerance, may help bacteria to develop resistance by providing alternative, faster evolutionary routes to resistance.^88^

Such non-genetic phenotype conversions have long been explained by shifts between stable configurations (“attractors”) of the gene regulatory network. But the involvement of fold-switching proteins in mutation-independent phenotypic switching represents additional versatile layer of non-genetic mechanisms that can have far reaching implications both in biology and medicine.

## Acknowledgments

P.K. thanks Dr. Sneha Bheemireddy for her thought comments on an initial version of the manuscript. Dr. Bhattacharya’s research performed in the Integrative Genomics Core at City of Hope was supported by 10.13039/100000054NCI grant P30CA033572. The content is solely the responsibility of the authors and does not necessarily represent the official views of the 10.13039/100000002NIH.

## Declaration of interests

The authors declare no competing interests.
